# Multi-volume rendering using depth buffers for surgical planning in virtual reality

**DOI:** 10.1007/s11548-025-03432-y

**Published:** 2025-06-07

**Authors:** Balázs Faludi, Marek Żelechowski, Maria Licci, Norbert Zentai, Attill Saemann, Daniel Studer, Georg Rauter, Raphael Guzman, Carol Hasler, Gregory F. Jost, Philippe C. Cattin

**Affiliations:** 1 Department of Biomedical Engineering, University of Basel, Basel, Switzerland; 2 Neurosurgery, University Children’s Hospital of Basel, Basel, Switzerland; 3 Neurosurgery, University Hospital of Basel, Basel, Switzerland; 4 Spinale Chirurgie, Hospital Biel, Biel, Switzerland

**Keywords:** Multi-volume rendering, Virtual reality, Surgical planning

## Abstract

**Purpose:**

Planning highly complex surgeries in virtual reality (VR) provides a user-friendly and natural way to navigate volumetric medical data and can improve the sense of depth and scale. Using ray marching-based volume rendering to display the data has several benefits over traditional mesh-based rendering, such as offering a more accurate and detailed visualization without the need for prior segmentation and meshing. However, volume rendering can be difficult to extend to support multiple intersecting volumes in a scene while maintaining a high enough update rate for a comfortable user experience in VR.

**Methods:**

Upon loading a volume, a rough ad hoc segmentation is performed using a motion-tracked controller. The segmentation is not used to extract a surface mesh and does not need to precisely define the exact surfaces to be rendered, as it only serves to separate the volume into individual sub-volumes, which are rendered in multiple, consecutive volume rendering passes. For each pass, the ray lengths are written into the camera depth buffer at early ray termination and read in subsequent passes to ensure correct occlusion between individual volumes.

**Results:**

We evaluate the performance of the multi-volume renderer using three different use cases and corresponding datasets. We show that the presented approach can avoid dropped frames at the typical update rate of 90 frames per second of a desktop-based VR system and, therefore, provide a comfortable user experience even in the presence of more than twenty individual volumes.

**Conclusion:**

Our proof-of-concept implementation shows the feasibility of VR-based surgical planning systems, which require dynamic and direct manipulation of the original volumetric data without sacrificing rendering performance and user experience.

**Supplementary Information:**

The online version contains supplementary material available at 10.1007/s11548-025-03432-y.

## Introduction

Using direct volume rendering (DVR) for surgical planning has several benefits compared to rasterization-based techniques. With DVR, the original raw volumetric data are retained and the images are generated directly from that data. This can result in higher-quality images with more visible object surface details, such as fine sutures or cracks, and better accuracy [1]. Generally, it also allows for an easy-to-use direct manipulation of the original data, e.g., to simulate tissue resection or deformation, without a loss of quality or volumetric information [2, 3].

One disadvantage of using a ray-marching-based volume renderer is the difficulty of extending it to support multiple, possibly even slightly overlapping, datasets in a single scene. This is especially a problem for VR applications where frame drops can cause motion sickness and achieving an acceptable rendering performance can be challenging even for a single volume. Although there are DVR-based medical visualization systems with VR support [4], these are limited to displaying a single volume at a time.

One possible approach to display multiple volumes simultaneously is to voxelize and resample the entire scene into a single volume which can then be rendered using any volume rendering technique [5]. This is a viable solution for specific use cases, such as a multimodal PET-CT scan, where multiple scalar volumes are acquired and stored in co-aligned scalar fields that do not need to be moved relative to each other [6, 7]. When relative movement of volumes is needed, which is a common requirement for many surgical planning scenarios, such as deformity correction and implant planning in spine surgery, the entire scene has to be resampled. This would incur a substantial amount of computation and preclude real-time interactions, which is needed for a planning system in VR. A further downside of this approach is a loss of image quality due to resampling and interpolation.

Another possible approach is to render all visible volumes in a single pass of the ray marching shader [8]. However, this requires loading all volumes into VRAM and modifying the ray marching implementation to consider all volumes at every ray marching step. This reduces cache hit rates and results in a lot of unavoidable branching in the shader, which has a severe negative impact on the rendering performance. Many optimizations have been suggested to try to mitigate these issues. Various techniques exist that subdivide the volume assembly into homogeneous regions where only a fixed number of volumes have to be considered, thereby simplifying the shader code and reducing the branching at the cost of added complexity and computation on the CPU [9]. However, these renderers have not been developed with VR applications in mind, and in our experiments, they do not perform well enough in that scenario, even when using high-end GPUs.

Recent advancements in neural networks and point-cloud-based methods have introduced 3D Gaussian splatting as a promising alternative to traditional mesh rendering or DVR [10]. This technique enables rendering highly realistic scenes in real-time. The main downsides of this approach are its need for extensive preprocessing and training for each new scene, poor support for real-time editing, and some difficulty to preserve fine details.

An alternative approach is to render each volume separately with multiple consecutive dispatches of the ray marching shader. The challenge with this approach is the correct handling of occlusion when volumes are allowed to overlap and intersect each other. This problem is similar to the rendering of intersecting meshes using rasterization. In the case of mesh rendering, common solutions include the painter’s algorithm or using depth buffers [11, 12].

**Fig. 1:**
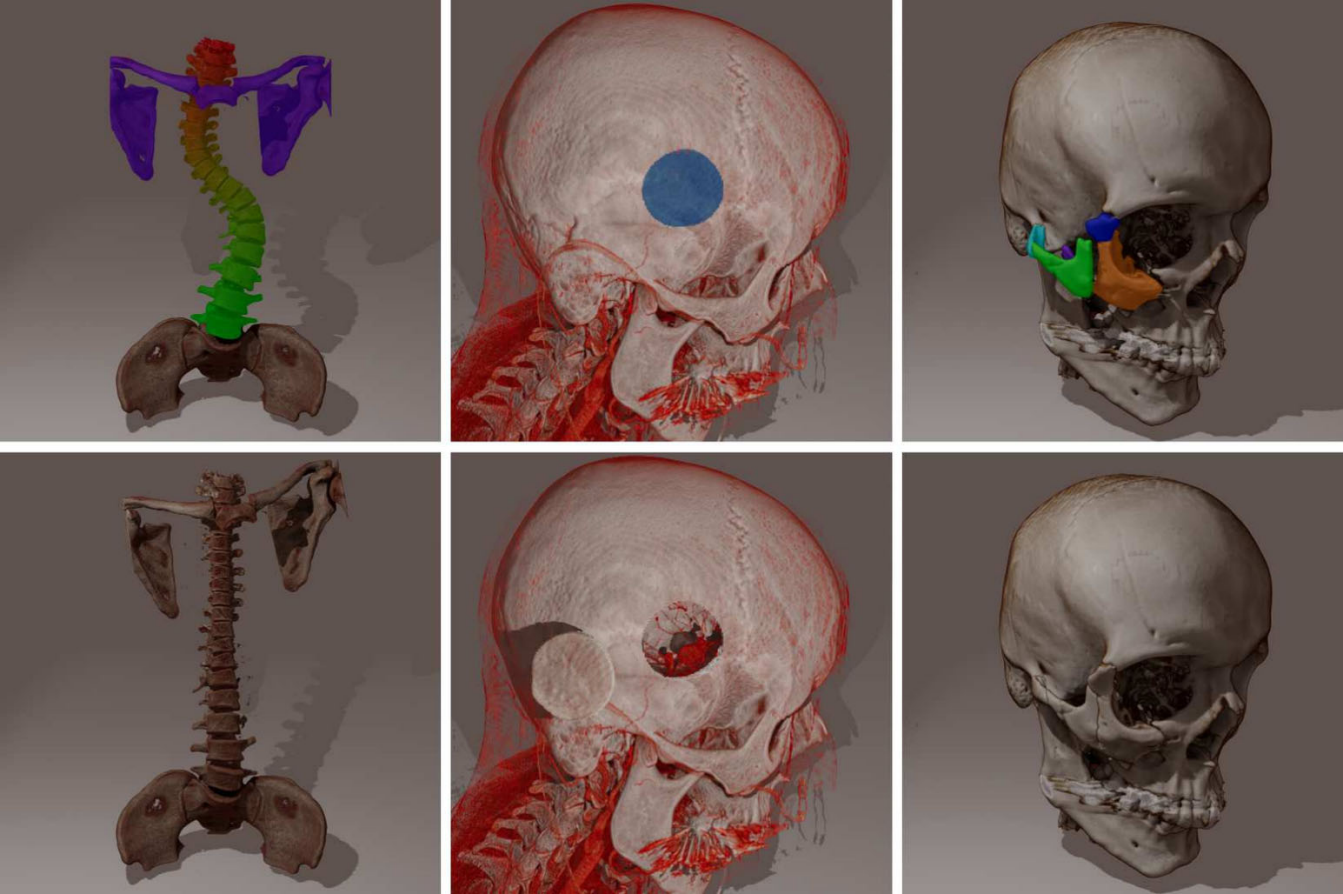
Three datasets rendered with our ray-marching-based multi-volume renderer. Left: Spinal fusion for scoliosis surgery. Middle: Sylvian keyhole approach for aneurysm clipping. Right: Zygomatic arch fracture reduction. The top row shows possible segmentations within each dataset. These segmentations can be created ad hoc, serve only to divide each dataset into sub-volumes, and are not needed for the visualization. The bottom row shows the same segments extracted into separate volumes and moved within the scene. The images serve as illustrations of the capabilities of the renderer and are not meant as an accurate representation of the shown procedures

In this work, we propose a novel multi-volume rendering approach capable of visualizing dozens of independent volume fragments with a performance suitable for surgical planning in VR. Although the performance of standalone VR systems is steadily increasing with each new generation of headsets, their performance is not yet adequate to achieve an acceptable level of image quality and rendering performance using ray marching. Therefore, for this project, we have been using a VR system that is directly connected to a desktop computer. We extend the single-volume case by using a depth buffer to correctly handle any number of intersecting volumes. Five experienced spine surgeons provided guidance, feedback and qualitative assessment for the development of this method. We explain the benefits of this approach over mesh-based rendering and evaluate the rendering performance for several use cases.

## Methods

The proposed method has been developed based on the suggestions and feedback of five practicing spine and neurosurgeons with an average of 17.6 years of professional experience, who are also co-authors of this work. The close collaboration with experienced clinicians ensured that the presented method has clear and meaningful benefits for a variety of use cases, such as visualization of complex anatomical structures [13], preparation and planning of minimally invasive cranial approaches [14], professional training of clinicians, and teaching medical students [15].

### Volume rendering

The volume renderer was developed as an extension to the built-in rendering pipeline of the Unity Engine 2022.1.21 (Unity Technologies, San Francisco, USA). This extension was created by attaching a separate command buffer to each relevant camera and light source in the scene and executing them after all opaque meshes in the scene have been rendered. Each command buffer contained a series of instructions to set up the necessary state and data before dispatching the ray marching shader. The ray marching process itself was implemented as a compute shader, with each thread processing a single ray. Multiple optimization methods, such as early ray termination, empty space skipping, and foveated rendering, have been included to achieve the required performance and interactivity needed for a surgical planning application in VR [16].

### Segmentation and extraction

**Fig. 2:**
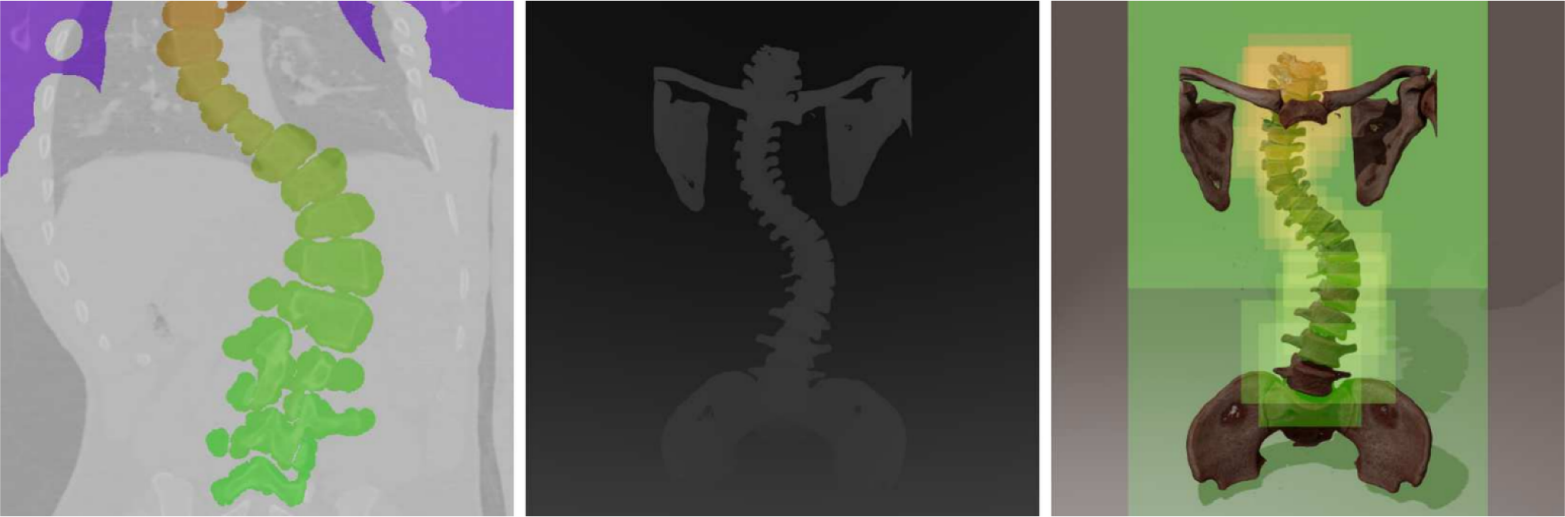
Left: Cross section through the scoliosis dataset showing that even a rough segmentation is sufficient to provide high-quality and accurate visualizations. Middle: The camera depth map used to ensure correct occlusion of the individual volumes (vertebrae). Right: Differently colored rectangles show the screen space bounds used to render each volume

For any volumetric dataset loaded into the visualization system, we optionally create an additional segmentation mask. This is a 3D texture of the same resolution as the volumetric scan data and a single 8-bit channel per voxel, allowing us to assign each voxel to one of 255 segments, where 0 means no assignment.

Segmenting specific organs, fragments of a bone fracture, and other regions of interest, can be done very efficiently and directly using motion-tracked controllers in VR. This can be used, for example, to interactively mark a portion of the cranial bone, as shown in Fig. 1 (middle) and Online Resource 1, to be removed during craniotomy and plan the optimal entry point and approach path to the pathological area. In contrast to the precise segmentations required for mesh-based rendering, we do not use the segments directly to visualize the object surfaces and merely need them to divide the datasets into sub-volumes.

Once a sub-volume has been roughly segmented, a new, independent volume has to be created containing the segmented data. To do this, the axis-aligned bounding box (AABB) of the segment is computed using an efficient compute shader. Once the AABB is known, a new volume is created by allocating a new 3D texture and copying the data from the original volume. Additional visualization properties, such as the selected transfer function or contrast settings, are also copied. Finally, the segment is hidden from the original volume by setting the segment opacity to 0, and the new volume is added to the scene.

### Multi-volume rendering

**Fig. 3:**
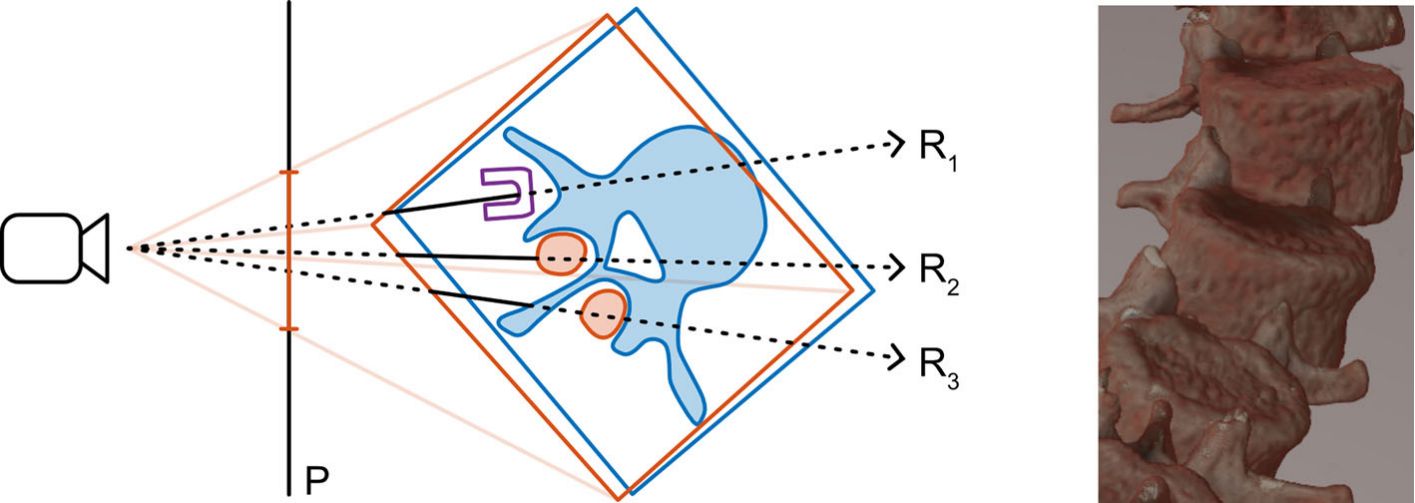
Left: An illustration of our method, simplified to a 2D cross section of a scene. The visible tissues and the bounding boxes of two adjacent vertebrae are shown in blue and orange. In the selected cross section, only the facet joints of the orange vertebra are visible. The projection of the orange bounding box onto the image plane *P* is illustrated. A mesh model of a pedicle screw head is shown in purple. Three exemplary rays $R_1$, $R_2$ and $R_3$ are shown. The segment of each ray that is processed by the ray marching shader while rendering the orange volume is indicated with a solid line. Right: An example of incorrect rendering without correct occlusion. Note that the transverse and articular processes of the inferior vertebrae are visible even though they should be occluded by the body of the superior vertebrae

Correct handling of occlusion is a challenging but important requirement for multi-volume rendering. Fig. 3 (left) shows an illustration of two partially overlapping volumes containing adjacent vertebrae of a spine. A single-pass volume renderer can produce a correct visualization of such a scene using a single dispatch of the ray marching shader and sampling all volumes at every ray marching step. However, this adds a lot of branching, complicates the execution flow of the shader, and thereby degrades the performance.

Instead, our approach processes the volumes one by one in an arbitrary order. A correct rendering of the scene in Fig. 3 cannot be achieved by independently rendering the two volumes into separate images and then using simple alpha blending without considering how the volumes occlude each other, as shown on the right. If the blue volume is rendered first, the orange facet joint traversed by ray $R_3$ would be visible through the blue spinous process instead of being occluded by it. If the orange volume is rendered first, the color of the orange facet joint at ray $R_2$ would be overwritten by the color of the blue vertebra. To sequentially render multiple volumes with correct occlusion, we need to store the distance to the camera at which each ray encountered something visible in the currently processed volume and clamp each ray to that distance when processing the next volume.

Therefore, we extended the single-volume case by computing the z-depth for all rays and writing it to a separate depth texture. The computation is simple and incurs no noticeable performance overhead since we can keep track of the length of the processed part of a ray during the ray marching process and output the distance to the camera if and when we reach the alpha threshold for early ray termination. If no visible voxel is encountered along a ray, the z-depth is set to the distance of the far plane. After the ray marching shader completes, the depth texture is blitted into the camera’s depth buffer with depth testing enabled. We do not write the depth texture directly in the ray marching shader because the pixel resolution of the camera and the resolution of our output may be different when foveated rendering is used. Fig. 2 (middle) shows the final depth buffer that was created during the rendering of a spine.

With the addition of writing out the depth of volume rendered objects, it is possible to render multiple volumes consecutively in any order and achieve correct rendering with occlusion, provided that the volumes use transfer functions that do not result in a significant number of semi-transparent voxels. If the blue volume in Fig. 3 is rendered first, the camera depth of the blue vertebra is written into the depth texture. During the subsequent rendering of the orange volume, the depth texture is sampled to limit the processing of ray $R_2$ to the depth of the spinous process, as indicated by the solid segment of the ray, thereby resulting in the correct occlusion of the orange facet joint. The process works equivalently in the opposite, or any arbitrary, order of volumes. Similarly, opaque mesh objects can be rendered before rendering any volumes to implement correct occlusion with mesh-based scene geometry, such as the pedicle screw head intersected by ray $R_1$ in Fig. 3.

In the single-volume case, dispatching a compute shader thread for every pixel in the output does not add much performance overhead compared to the heavy workload of the ray marching process itself. If the single volume covers a big portion of the field of view, most of the frame time will be consumed by marching the rays and sampling the volume data, and therefore, saving a few unnecessary shader invocations and subsequent blits does not result in a noticeable improvement in performance. If the single volume is far away from the camera or has been scaled down to only cover a small portion of the final image, the ray marching workload will be reduced considerably, and the overhead of a single, mostly unnecessary full-screen blit is negligible on modern graphics devices. However, in the multi-volume case, each additional volume results in an extra dispatch of the ray marching shader. If we do not limit the number of pixels processed by each dispatch, the GPU utilization will suffer and the rendering performance will be noticeably reduced. We alleviate this issue by projecting the eight corners of each volume into the screen space of the camera and determine the minimum bounding rectangle. This is illustrated in Fig. 3 and an example is visualized in Fig. 2 (right). The ray marching shader is then dispatched only for the pixels within the screen space bounding rectangle of each volume.

## Results

### Performance evaluation

For this method to be feasible for surgical planning in VR, it has to perform fast enough, even with potentially dozens of individual volumes in the scene. We prepared several multi-volume configurations to evaluate the rendering performance and to measure the impact of extracting parts of a volume into new volumes. For each test, we load the volumes, wait for a one second warm-up period, and then render the scene while the loaded volumes complete two full rotations around the vertical world axis. We measure the frame delta times for 10 s with vertical sync disabled. Standard early ray termination was enabled and foveated rendering was disabled, which is a common configuration for volume rendering performance measurements. The image resolution was set to 2160 $\times$ 2160 for both eyes, which corresponds to the native resolution of the HP Reverb G2 HMD (HP Inc., Palo Alto, USA). The dataset resolutions for each test are specified in Table 1. The tests were conducted on a workstation with an AMD Ryzen 9 5900X 12-Core CPU @ 3.70 GHz, an Nvidia GeForce RTX 4080 GPU (driver version 531.18), 32 GB RAM, running Windows 11 (22H2).

**Fig. 4:**
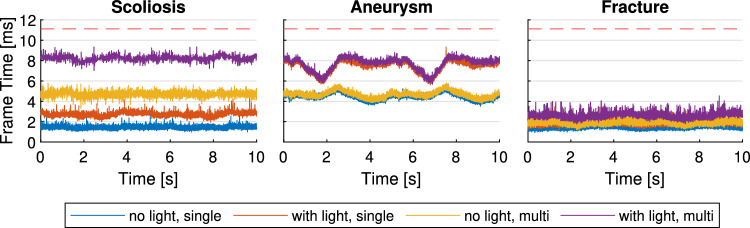
Frame times in ms while rendering 3 different datasets completing 2 full rotations around the vertical axis in 10 s. For each dataset, the performance is shown with/without a real-time light source and shadow map, and with/without extracted segments, i.e., multi-volume rendering. The red dashed line shows the frame time budget corresponding to 90 Hz (11.1 ms) required for smooth virtual reality rendering

### Rendering performance

We used 3 anonymized CT datasets, from the University Hospital of Basel, which are shown in Fig. 1, to measure the performance of the multi-volume renderer.

- **Scoliosis.** In this dataset, we removed the ribs from the dataset for better visibility of the vertebrae and created 20 segments (C6, C7, T1–T12, L1–L5, and a single segment for the clavicles and scapula), resulting in a total of 21 individually movable volumes. This case represents the most challenging and complex cases that we expect a multi-volume renderer to be used for, as no other type of surgery is likely to require moving significantly more bones than spinal fusion surgery.
- **Aneurysm.** In this case, we created a circular segment through the side of a skull as an approximation of a minimally invasive craniotomy (Sylvian keyhole approach) for aneurysm surgery, resulting in a total of 2 volumes. This example has been selected to represent the simplest use case of extracting and moving a single additional volume.
- **Fracture.** A skull with a fracture of the zygomaticomaxillary complex, where we segmented 5 bone fragments for a total of 6 volumes. This dataset represents the typical scenario of a few volumes that need to be adjusted to reduce a fracture.

Fig. 4 shows the rendering performance for three different datasets, each with four conditions. We measured the performance of the single-volume case without any extracted segments. We compare this to the performance of the multi-volume case, where we segmented parts of the dataset and extracted all segments into separate volumes. We also repeated the same measurements with a single real-time point light source in the scene, which requires an additional depth-only ray-marching pass to update its shadow map. The average frame rates of all benchmarks are shown in Table 1.

**Table 1:** The average frame rate in Hz for each dataset and configuration. Static refers to no real-time lighting, while dynamic means a single real-time light source. The resolution indicates the voxel count of the original dataset before segmentation

	Resolution	Volumes	Single-Volume		Multi-Volume	
			Static	Dynamic	Static	Dynamic
Scoliosis	$512 \times 512 \times 775$	21	736	379	214	122
Aneurysm	$512 \times 512 \times 727$	2	229	136	219	132
Fracture	$512 \times 512 \times 485$	6	739	581	551	397

## Discussion and conclusions

We demonstrated the feasibility of ray-marching-based multi-volume rendering for surgical planning in VR. As we mentioned in the introduction, standalone VR systems that are currently available on the market are not powerful enough to visualize volumetric medical data of typical resolution and configuration using a ray-marching-based volume renderer. We expect this to change with the next couple of generations of standalone headsets, but for the time being, the focus of this project was on HMDs tethered to a desktop computer.

During our measurements, the frame rate consistently stayed above 90 Hz, which is the native refresh rate of many contemporary HMDs. The performance overhead correlates with the number, size and complexity of volumes that are within the viewing frustum. Extracting the segmented regions resulted in a frame rate drop of 71 % for the scoliosis dataset (20 vertebrae), 25 % for the fracture case (5 bone fragments), and a negligible 4 % for the aneurysm case when compared to rendering the whole dataset at once.

The noticeable performance increase around seconds 2 and 5 while rendering the aneurysm dataset with real-time lighting enabled was likely caused by the noisiest and computationally challenging areas of the volume being hidden behind the skull from the light’s point of view.

As shown in Fig. 2 (right), we limit the rendering area to the screen space bounds of each volume. This could be further enhanced by switching from the compute shader implementation to a fragment shader and relying on the rasterizer to invoke the ray marching process only on fragments generated for a bounding box or convex hull of the volume.

An extracted segment is only hidden from the original volume by setting its opacity to 0. Therefore, these areas cannot be skipped efficiently when marching through the original volume. A possible improvement would be to bake the removal into the original volume and update the empty space skipping data. This would require reloading the document or keeping an unmodified copy of the volume data in memory in case the user wants to undo the extraction. Alternatively, the empty space skipping data could take into account the opacity of all segments. This would require updating this data whenever a segment’s opacity changes, resulting in a temporary but noticeable performance hit.

Currently, the rendering order of the volumes is the order of creation. Ordering the volumes by distance to the camera or by hierarchy, i.e., rendering extracted volumes before their origin volume, may result in better performance in some cases due to depth testing.

**Table 2:** Comparison of different approaches for rendering multiple intersecting volumes

Approach	Occlusion	Main Advantages and Limitations
Multi-Pass with	Per pixel	Good performance even with over 20 volumes
Z-Buffer (Ours)	Depth test	Allows free volume rearrangement and TF editing
		Transparency not well supported
Mesh-Model	Per pixel	Good performance; mainly depends on polygon count
Rasterization	Depth test	Requires segmentation and manual verification
		Transparency and TF editing not well supported
One-Pass Ray	Implicit	Poor performance even with low volume count
Marching		No preprocessing needed and TF editing supported
		Volume count limited by VRAM and shader support
Single Merged	Implicit	Good performance; comparable to a single volume
Volume		Requires resampling after moving a volume
		Resampling can cause loss of image quality
3D Gaussian	Front-to-back	Decent performance and realistic rendering
Splatting	Sorted Gaussians	Dynamic scenes and TF editing not well supported
		Extensive preprocessing for each scene

Table 2 summarizes the strengths and limitations of different approaches to visualizing multiple volumetric medical images in a scene. Our method has comparable limitations to a mesh-based approach, wherein visualizing semi-transparent regions is not well supported. However, our approach provides several advantages, leveraging the more dynamic and flexible nature of volume rendering. A rough, ad hoc segmentation, as shown in Fig. 2 (left), is sufficient to be able to select and move parts of a volume since the exact shape and visual appearance of tissues depends only on the raw data. This is especially beneficial in the case of bone fractures, where automated segmentation of individual bone fragments is still challenging. Additionally, the full volumetric information is retained while moving sub-volumes, which is essential for planning tasks such as pedicle screw placement. This enables the implementation of more realistic and consistent planar clipping through the dataset and other features that rely on real-time modifications to the volumetric data, such as resection of tissues or planning and simulating bone cuts. Mesh rendering does not fulfill the necessary criteria in these cases, as it does not preserve the volumetric data and requires high-quality segmentation and meshing upfront.

One-pass ray marching solutions, where all relevant volumes are sampled at each step, retain the main advantages of DVR, support the real-time editing of transfer functions (TF), and allow a free rearrangement of any volume. Compared to a one-pass ray marching solution, our approach avoids the performance penalties caused by the increased complexity and branching in the one-pass shader. In our early experiments, even scenes with as few as 2–4 volumes could not be rendered at 90 Hz using a simple one-pass approach without acceleration structures. Since 3D texture arrays are not a commonly supported data structure, indexing into a list of volumes requires a more complex setup involving either branching in the shader, merging volumes using different texture channels, stacking multiple volumes side by side or other workarounds. These workarounds are more likely to run into limitations of the hardware and graphics libraries, such as a maximum of 2 GB per texture, a maxiumum of 2048 voxels per axis, or the available VRAM. A multi-pass approach, such as ours, is less likely to encounter these problems, since the volumes are processed sequentially.

Resampling and merging multiple volumes into a single combined volume can improve rendering performance compared to the one-pass approach. This approach reduces shader complexity and simplifies the rendering pipeline, with the main remaining overhead being the additional texture sampling and blending at each step. However, resampling can degrade the image quality, and any change to the spatial arrangement of the volumes requires repeating the merging step. Further, when combining data from different modalities, the transfer functions may need to be applied before merging which further limits the flexibility and interactive control.

Finally, 3D Gaussian splatting enables real-time visualization of static scenes with high visual fidelity using a neural-network-based rendering approach. However, it has several downsides limiting its usability for surgical planning. The training process would require as input a set of pre-rendered images of the scene from a variety of viewpoints, generated using an alternative rendering method. It would also require retraining the network for each dataset and does not have good support for dynamic scenes or moving objects, thereby making it challenging, to freely move parts of a dataset or to dynamically adapt the transfer function. Although recent work has introduced support for animation and deformation, precise real-time interactions still pose a challenge. These factors make the approach unsuitable for surgical planning tasks, where individual structures need to be manipulated directly and interactively.

Visualizations of medical datasets are often limited in flexibility and primarily involve inspecting static volumetric scans of the patient’s anatomy. Making the visualization more dynamic and adding the possibility to rearrange and modify the data in real-time opens new possibilities. Combined with the direct and intuitive interactions that can be achieved with the motion-tracked controllers of a VR system, the preparation and planning of surgical procedures could be improved in terms of time, accessibility, and adoption. Although no formal clinical study was conducted in this work, the proposed method was developed in collaboration with experienced neurosurgeons to address real-world planning needs, providing a foundation for future quantitative evaluation.

## Supplementary Information

Below is the link to the electronic supplementary material.

Supplementary file 1 (mp4 407801 KB)

Supplementary file 2 (pdf 110 KB)

## Data Availability

This article is accompanied by a supplementary video illustrating the main use cases of the presented approach and a supplementary document listing the relevant code excerpts of the rendering pipeline.
