# Supplementary material for: Multi-volume rendering using depth buffers for surgical planning in virtual reality
Source: Int J Comput Assist Radiol Surg. 2025 Jun 7;20(11):2251–8. doi: 10.1007/s11548-025-03432-y (PMC12575470; doi:10.1007/s11548-025-03432-y)
Supplement: Supplementary file 2 — (pdf 110 KB) [file 11548_2025_3432_MOESM2_ESM.pdf]

Multi-Volume Rendering using Depth Buffers for Surgical Planning in Virtual Reality

Supplementary Material

Balázs Faludi[1*], Marek Żelechowski[1], Maria Licci[2, 3], Norbert Zentai[1], Attill Saemann[3], Daniel Studer[2], Georg Rauter[1], Raphael Guzman[2, 3], Carol Hasler[2], Gregory F. Jost[4], Philippe C. Cattin[1]

[1*] Department of Biomedical Engineering, University of Basel, Switzerland .
[2] Neurosurgery, University Children's Hospital of Basel, Switzerland .
[3] Neurosurgery, University Hospital of Basel, Switzerland .
[4] Spinale Chirurgie, Hospital Biel, Switzerland .

*Corresponding author(s). E-mail(s): balazs.faludi@unibas.ch;

Implementation Details

In this section we show the main integration points and relevant code excerpts of our multi-volume rendering pipeline. Some details that are irrelevant to the main focus of this work are omitted for brevity.

As explained in the manuscript, our volume renderer is built in the Unity Engine and extends the built-in rendering pipeline using `UnityEngine.CommandBuffer` objects. For every camera in the scene, and optionally every light source, we create and maintain a command buffer. The following excerpt shows how each command buffer is

updated every frame, to schedule the right graphics commands to be executed during the rendering pass of the engine.

```
public void UpdateCommandBuffer(ReadOnlyList<Volume> volumes)
{
    // Set up general shader state
    CommandBuffer.Clear();
    CommandBuffer.SetKeyword(shader, _multiVolumeShaderKeyword,
       volumes.Count > 1);

    // Set the depth map, so we can use it to skip rays that are
    // blocked by close objects.
    CommandBuffer.SetComputeTextureParam(
        RayMarchShader,
        KernelIndex,
        CameraDepthTexturePropertyID, // _CameraDepthTexture
        BuiltinRenderTextureType.Depth);

    for (int i = 0; i < volumes.Count; i++)
    {
        var volume = volumes[i];
        PrepareVolume(volume);
        RenderVolume(volume);
    }
    PostRender();
}
```

We iterate over the list of volumes in no specific order and schedule commands to update texture, constant buffer and shader parameter state in the `PrepareVolume` method and dispatch the actual ray marching shader in the `RenderVolume` method.

In the `PrepareVolume` method, the necessary state is set via the command buffer of the rendering target (a camera or light source).

```
void PrepareVolume(Volume volume)
{
    var voxelData = volume.VoxelData;
    var buffer = CommandBuffer;
    var shader = RayMarchShader;
    var whiteTexture3D = DefaultTexture3D.WhiteTexture;

    var transferTexture = voxelData.TransferFunction?.Texture;
    var volumeResolution = TypeHelper.ToVector4(voxelData.Resolution);
    var volumeVoxelSize = TypeHelper.ToVector4(voxelData.VoxelSize);
    var slopeIntercept = TypeHelper.ToVector4(voxelData.TextureToTransferFunction);
    var distanceThreshold = TypeHelper.ToVector4(voxelData.DistanceThreshold);
    var voxelStepSize = new RangeFloat(1, 4);
    float stepSize = Mathf.Min(1.0f / 32.0f,
        voxelData.StepSizeScale * voxelStepSize.Lerp(1 - Quality));

    // Data textures. These are managed and updated asynchronously
    // by a resource system as needed.
    buffer.SetComputeTextureParam(shader, KernelIndex,
        ShaderProperties.TransferFunctionID, transferTexture); // shared
    buffer.SetComputeTextureParam(shader, KernelIndex,
        ShaderProperties.VoxelDataID, volume.VoxelDataTexture);
```

```
    buffer.SetComputeTextureParam(shader, KernelIndex,
        ShaderProperties.DistanceID, volume.DistanceMapTexture);
    buffer.SetComputeTextureParam(shader, KernelIndex,
        ShaderProperties.OcclusionID, volume.OcclusionTexture);

    // General volume configuration.
    buffer.SetComputeFloatParam(shader,
        ShaderProperties.StepSizeID, stepSize);
    buffer.SetComputeVectorParam(shader,
        ShaderProperties.DistanceThresholdID, distanceThreshold);
    buffer.SetComputeVectorParam(shader,
        ShaderProperties.ShadowFactorsID, 1);
    buffer.SetComputeVectorParam(shader,
        ShaderProperties.VolumeResolutionID, volumeResolution);
    buffer.SetComputeVectorParam(shader,
        ShaderProperties.VolumeVoxelSizeID, volumeVoxelSize);
    buffer.SetComputeVectorParam(shader,
        ShaderProperties.SlopeInterceptID, slopeIntercept);
}
```

Once the commands for updating shader parameters and volume data have been scheduled, the ray marching shader can be dispatched for the volume. For our custom foveated rendering implementation, we dispatch multiple renders with different resolutions. As mentioned in the manuscript, foveated rendering was disabled during performance measurements.

```
void RenderVolume(Volume volume)
{
```

```
if (!UpdateRenderBounds(volume))
    return;

UpdateVoxelObjectParameters(volume);
UpdateShadowMapParameters(volume);
UpdateFoveaLevels();

while (FoveatedSampleLevel.Count < FoveaLevels.Length)
    FoveatedSampleLevel.Add("Render Foveated Level "
        + $"{FoveatedSampleLevel.Count}");

float scale = AppSettings.ResolutionScaleType.Value
    == ResolutionScaleType.VolumeOnly
    ? AppSettings.ResolutionScale.Value : 1;

for (int i = FoveaLevels.Length - 1; i >= 0; i--)
{
    // Update fovea dependent parameters.
    UpdateFoveaParameters(i == 0 ? 0 : FoveaLevels[i - 1].Radius,
        FoveaLevels[i].Radius, FoveaLevels[i].Sampling * scale);
    UpdateDepthNormalsParameters();

    // Schedule the execution of the volume shader and subsequent
    // copying of the results into the target image.
    DispatchShader(volume);
    OnPostBlitResult();
```

```
    }
}
```

Finally, the ray marching shader is dispatched to render into a temporary target texture, which is then blitted into the final image.

```
void DispatchShader()
{
    var eyeResolution = CurrentFoveaRing.Resolution.ToVector2Int();

    // Compute screen space bounds for the shader dispatch.
    var renderBoundsNormalized = CurrentRenderBounds;
    var renderBoundsPixel = new BoundsInt
    {
        min = new Vector3Int(
            Mathf.FloorToInt(renderBoundsNormalized.min.x
                * CurrentFoveaRing.Resolution.x),
            Mathf.FloorToInt(renderBoundsNormalized.min.y
                * CurrentFoveaRing.Resolution.y)),
        max = new Vector3Int(
            Mathf.CeilToInt(renderBoundsNormalized.max.x
                * CurrentFoveaRing.Resolution.x),
            Mathf.CeilToInt(renderBoundsNormalized.max.y
                * CurrentFoveaRing.Resolution.y))
    };
    var resolution = (Vector2Int)renderBoundsPixel.size;

    // Determine the number of thread groups we have to dispatch
```

```
// to render the entire texture.
var threadGroupCount = ((Vector2)resolution)
    .DivideBy(ThreadGroupSize).Ceil().ToVector2Int();
if (threadGroupCount.x < 1 || threadGroupCount.y < 1)
{
    Debug.LogWarning("VolumeRenderer cannot render. "
        + "Thread group size should be > 0.");
    return;
}

// Determine the target texture resolution, based on stereo rendering mode.
bool stereoEnabled = _camera.stereoEnabled;
var fullResolution = _camera.pixelRect.size;
var pixelOffset = renderBoundsPixel.min;
EyeResolutionInt2[0] = eyeResolution.x;
EyeResolutionInt2[1] = eyeResolution.y;
EyeResolutionInt2[2] = (int)pixelOffset.x;
EyeResolutionInt2[3] = (int)pixelOffset.y;
var targetResolution = resolution;
if (stereoEnabled && XRSettings.stereoRenderingMode
    == XRSettings.StereoRenderingMode.SinglePass)
{
    targetResolution.x *= 2;
}

// Prepare target render texture descriptor.
var targetDescriptor = new RenderTextureDescriptor(
```

```
    targetResolution.x, targetResolution.y)
{
    colorFormat = RenderTextureFormat.ARGBFloat,
    depthBufferBits = 0,
    msaaSamples = 1,
    enableRandomWrite = true,
    dimension = stereoEnabled
        ? XRSettings.eyeTextureDesc.dimension
        : TextureDimension.Tex2D,
    volumeDepth = stereoEnabled
        ? XRSettings.eyeTextureDesc.volumeDepth
        : 1,
    vrUsage = stereoEnabled
        ? XRSettings.eyeTextureDesc.vrUsage
        : VRTextureUsage.None,
};

// Render the volume into a temporary render texture.
CommandBuffer.GetTemporaryRT(ResultPropertyID,
    targetDescriptor, FilterMode.Bilinear);
CommandBuffer.SetComputeTextureParam(RayMarchShader,
    KernelIndex, ResultPropertyID, TargetIdentifier);
CommandBuffer.SetComputeIntParams(RayMarchShader,
    EyeResolutionID, EyeResolutionInt2);
CommandBuffer.SetComputeFloatParam(RayMarchShader,
    FoveaSamplingID, CurrentFoveaRing.Sampling);
```

```
// Render the left eye
if (ShouldRenderEye(MonoOrStereoscopicEye.Left))
{
    CommandBuffer.SetComputeIntParam(RayMarchShader, "EyeIndex", 0);
    UpdateCameraParameters(_camera.stereoEnabled
        ? MonoOrStereoscopicEye.Left
        : MonoOrStereoscopicEye.Mono);
    CommandBuffer.DispatchCompute(RayMarchShader,
        KernelIndex, threadGroupCount.x, threadGroupCount.y, 1);
}

// Render the right eye
if (ShouldRenderEye(MonoOrStereoscopicEye.Right))
{
    CommandBuffer.SetComputeIntParam(RayMarchShader, "EyeIndex", 1);
    UpdateCameraParameters(MonoOrStereoscopicEye.Right);
    CommandBuffer.DispatchCompute(RayMarchShader,
        KernelIndex, threadGroupCount.x, threadGroupCount.y, 1);
}

// Set fovea parameters for the blit material.
CommandBuffer.SetGlobalVector(FoveaRingID, CurrentFoveaRing.ShaderParameter);
CommandBuffer.SetGlobalVector(ResolutionID, fullResolution);
CommandBuffer.SetGlobalVector("DestBounds", new Vector4(
    (float)renderBoundsPixel.min.x / eyeResolution.x,
    (float)renderBoundsPixel.min.y / eyeResolution.y,
    (float)renderBoundsPixel.size.x / eyeResolution.x,
```

```
        (float)renderBoundsPixel.size.y / eyeResolution.y));
    CommandBuffer.SetGlobalVector("DestBoundsPx", new Vector4(
        renderBoundsPixel.min.x,
        renderBoundsPixel.min.y,
        renderBoundsPixel.size.x,
        renderBoundsPixel.size.y));

    // Blit the result into the target.
    int blitMaterialPass = CameraBlitID;
    var target = BuiltinRenderTextureType.CameraTarget;
    CommandBuffer.Blit(TargetIdentifier, target, BlitMaterial, blitMaterialPass);
    CommandBuffer.ReleaseTemporaryRT(ResultPropertyID);
}
```
